# Estimating the risk of Dengue, Chikungunya and Zika outbreaks in a large European city

**DOI:** 10.1038/s41598-018-34664-5

**Published:** 2018-11-06

**Authors:** Angelo G. Solimini, Mattia Manica, Roberto Rosà, Alessandra della Torre, Beniamino Caputo

**Affiliations:** 1 Department of Public Health and Infectious Diseases, Universita’ La Sapienza, Rome, Italy; 2 Department of Biodiversity and Molecular Ecology, Research and Innovation Centre, Fondazione Edmund Mach, San Michele all’Adige, TN Italy

## Abstract

Outbreaks of arbovirus infections vectored by invasive *Aedes albopictus* have already occurred and are predicted to become increasingly frequent in Southern Europe. We present a probabilistic model to assess risk of arbovirus outbreaks based on incident cases worldwide, on the probability of arrival of infected travelers, and on the abundance of the vector species. Our results show a significant risk of Chikungunya outbreak in Rome from mid June to October in simulations with high human biting rates (i.e. when ≥50% of the population is bitten every day). The outbreak risk is predicted to be highest for Chikungunya and null for Zika. Simulated increase of incident cases in selected endemic countries has no major impact on the outbreak risk. The model correctly estimated the number of imported cases and can be easily adapted to other urban areas where *Ae*. *albopictus* is the only potential vector present.

## Introduction

Arboviruses (arthropod-borne viruses) are neglected human pathogens responsible of a large burden of morbidity especially in tropical and subtropical regions where mosquito species are abundant^1^. In the last years, arboviruses such Dengue (DENV), Chikungunya (CHIKV) and Zika (ZIKV) extended their geographical range allowing autochthonous transmission and causing major outbreaks at temperate latitudes in almost all continents^2^. In Europe, recent DENV outbreaks were recorded in France^3,4^ and Croatia^5^ while outbreaks of CHIKV were recorded in France^6^ and Italy^7,8^. Not surprisingly, all these events took place in Southern Europe (Euro-Mediterranean region), where a stable and extensive colonization by the Asian tiger mosquito *Aedes albopictus* creates permissive conditions for transmission. *Aedes albopictus* is less efficient in DENV and ZIKV transmission compared to *Ae*. *aegypti*^9,10^ while in many locations worldwide both *Ae*. *aegypti* and *Ae*. *albopictus* are generally highly susceptible to infection, dissemination and transmission of CHIKV^11^. Alarmingly, *Ae*. *albopictus* may reach high densities in urban areas of many Mediterranean countries^12^, reaching values up to 40 mosquito females per person^13^.

The establishment of a competent vector and suitable conditions for secondary transmission are both necessary to cause an outbreak *in case* of introduction of the virus from an external source. Hence, an increase of incident cases in tropical/subtropical areas coupled with increased traveller flow are critical factors in order to catalyze local outbreaks in Euro-Mediterranean cities. Unfortunately, the risk of outbreak cannot be easily predicted. In this context, mathematical and statistical models are powerful tools to elucidate the role played by each factor in determining the likelihood of local transmission^14^ and are often the only choice left to health authorities when facing the problem of calculating the outbreak risk.

Local-scale models are availble to assess the risk of outbreak probability for DENV, CHIKV and ZIKV in temperate regions following the importation of a single infectious case^15,16^. However, model predictions are based on data of *Ae*. *albopictus* abundance in Northern Italy without estimates of the probability of imported cases from abroad. On the other hand, the large-scale model developed by^17^ assesses the seasonal risks of the introduction and spread of ZIKV in Europe without vector abundance data.

Here we present a probabilistic model to assess the risk of CHIKV, DENV and ZIKV outbreaks in Rome based on actual data on the abundance of the vector species, *Ae. albopictus,* as well as on estimates of risk of virus introduction via the large flow of travelers through the international airport of Rome Fiumicino (FCO).

## Materials and Methods

### Model

Following the approach of Johansson and colleagues^18–20^, a probabilistic model was developed to compute the probability of observing an mosquito-borne arbovirus outbreak (*P*) in Rome generated by the importation of infected human cases. This probability is the result of two main processes, i.e. the arrivals of infected travelers to Rome and the autochthonous transmission of a specific arbovirus within the city. The explicit formula is the following:

1 $$P=1-{\prod }_{j}{\prod }_{i}{\prod }_{t}{(1-{\rho }_{ijt}+{\rho }_{ijt}\frac{{R}_{0,i}^{VH}+1}{{R}_{0,i}^{HV}({R}_{0,i}^{HV}+1)})}^{{I}_{ijt}}$$

The probability of outbreak in Rome (Eq. 1) could be interpreted as one minus the probability that no outbreak would take place. The latter is the product, for each country *j*, week *i* and type of traveler *t* (resident or visitor, see below for details), of the sum of probability of not arriving in Rome $1-{\rho }_{ijt}$ and the probability that one arrives without generating any autochthonous transmission ${\rho }_{ijt}({R}_{0,i}^{VH}+1)/({R}_{0,i}^{HV}({R}_{0,i}^{HV}+1))$, to the power of the number of infected host ${I}_{ijt}$ (see details in Technical Appendixes, section 1).

The model was run over an ensemble of 100,000 parameter sets, each one obtained by resampling their specific distribution (Table 1), in order to take into account the uncertainty. The results are reported as mean probability and credible interval per week. Additionally, we computed the year cumulative probability of outbreak, the expected cumulative number of incoming infected hosts in Rome and the frequency of infected host among travelers from different WHO regions.

**Table 1:** Distributions and mathematical equations used to estimate parameters in the calculation of the basic reproductive number (*R*_0_) for DENV, CHIKV and ZIKV.

Parameter	Description	Virus	Specification	Value	Reference
*m*	Mosquito mortality rate (1 /m = average mosquito life-span in days)	Dengue	Function	2(0.031 + 95820e^(T - 50.4)^)	Manica *et al*. 2017
		Chikungunya	Function	2(0.031 + 95820e^(T - 50.4)^)	Manica *et al*. 2017
		Zika	Function	2(0.031 + 95820e^(T - 50.4)^)	Manica *et al*. 2017
*χ*_*H*_	Susceptibility to infection of humans, transmission efficiency from an infected mosquito to human	Dengue	Uniform	Min = 0.56; Max = 0.67	Vega-Rua *et al*. 2013
		Chikungunya	Uniform	Min = 0.14; Max = 0.84	Vega-Rua *et al*. 2013
		Zika	Uniform	Min = 0.19; Max = 0.39	Di Luca *et al*. 2016
*χ*_*H*_	Susceptibility to infection of mosquito, transmission efficiency from an infected human to mosquito	Dengue	Uniform	Min = 0.14; Max = 0.39	Talbalaghi *et al*. 2010
		Chikungunya	Uniform	Min = 0.75; Max = 0.90	Talbalaghi *et al*. 2010
		Zika	Uniform	Min = 0.03; Max = 0.17	Di Luca *et al*. 2016
1/*ω*_*V*_	Length of extrinsic incubation period (days)	Dengue	LogNormal	Mean = 2.9–0.08 T, SD = sqrt(1/4.9)	Chan & Johansson 2012
		Chikungunya	Uniform	Min = 2; Max = 3)	Dubrulle *et al*. 2009
		Zika	Uniform	Min = 7; Max = 14)	Guzzetta 2016 *et al*. 2016
1/*γ*	Infectious period in human hosts (days)	Dengue	Uniform	Min = 2; Max = 7 [Accessed 2017/02/27]	http://www.who.int/denguecontrol/human/en/
		Chikungunya	Uniform	Min = 2; Max = 6 [Accessed 2017/02/27]	https://wwwnc.cdc.gov/travel/yellowbook/2016/infectious-diseases-related-to-travel/chikungunya
		Zika	Uniform	Min = 4; Max = 7	Guzzetta *et al*. 2016
*k*	Biting rate	0.09	Uniform	Min = 0.08; Max = 0.1	Manica *et al*. 2017
	Reporting rate	Dengue		22%	Shepard *et al*. 2014
		Chikungunya		54%	Moro *et al*. 2010
		Zika		10%	Zhang *et al*. 2017
	Symptomatic rate	Dengue		24%	Shepard *et al*. 2014
		Chikungunya		84%	Moro *et al*. 2010
		Zika		20%	Zhang *et al*. 2017
*T*	Mean Temperature	—		Mean and SD from air temperature in Rome 2003–2014 (°C)	Meteorological weather station “Roma sud” Hydrographic Service of Regione Lazio (http://www.idrografico.roma.it/annali).
*V*/*H*	Vector to host ratio	—		Set according to different scenario	This work

Model parametrization of the mosquito-borne arbovirus autochthonous transmission and the probability of arrival of infected travelers are described below and detailed in Table 1 as well as in Technical Appendixes (sections 3 and 4).

### Autochthonous transmission

Autochthonous transmission is described on the basis of the basic reproduction number (*R*_0_) that in the case of mosquito-borne pathogen is computed as the product between the number of infectious mosquitoes generated from an infectious human (*R*_*0*_^*HV*^) and the number of infectious humans generated by the infectious mosquitoes surviving the extrinsic incubation period (*R*_*0*_^*VH*^), as follows (see^21,22^):

2 $${R}_{0}={R}_{0}^{VH}{R}_{0}^{HV}=\frac{k{\chi }_{H}}{m}\frac{k{\chi }_{V}}{\gamma }\frac{V}{H}\frac{{\omega }_{V}}{{\omega }_{V}+m}$$

When *R*_0_ < 1 (epidemic threshold), the probability of observing sustained autochthonous transmission after importation of a single case is negligible. On the other hand, when *R*_0_ > 1 the probability of observing sustained autochthonous arbovirus transmission after importation of a single case is

3 $$1-\frac{{R}_{0}^{VH}+1}{{R}_{0}^{HV}({R}_{0}^{HV}+1)}$$

as demonstrated by^23^.

*R*_0_ in (2) depends on several eco-epidemiological parameters related to the mosquito-borne arbovirus transmission and the mosquito life cycle that were defined following recent literature on Italian *Ae*. *albopictus* (Table 1). For every simulation run, every parameter value was sampled from its specific distribution, unless otherwise specified (Table 1). Temperature-dependence of some parameters (such as mosquito mortality) was incorporated using weekly temperatures recorded in Rome form meteorological stations (for details see Technical Appendixes, section 2).

Precise and reliable estimates of mosquito abundance through time over a large metropolitan area like Rome are challenging to obtain. However, we relied on previous field experiments^24,25^ and to recent model results^13,26^ to get an estimate of peaking mosquito abundance. Additionally, the temporal dynamic vector abundance was described as a function of time using previously collected empirical data^25^ (see Fig. S1 in Technical Appendixes).

On this basis, under the hypothesis of homogeneous mixing, we derived *R*_0_ for DENV, CHIKV and ZIKV for three scenarios of human biting rate (HBR = 0.1; 0.5; 1) corresponding respectively to 10%, 50% and 100% of human hosts bitten every day (Technical Appendixes, section 3).

### Probability of arrival of infected travelers

The expected number of CHIKV, DENV and ZIKV infected travelers arriving in Rome can be derived from the weekly probability of traveling to Rome, for each selected country, and the weekly number of incident cases recorded in the same country. The number of infected travelers to Rome is assumed to follow a binomial distribution of parameter *ρ* (probability of traveling to Rome) and size *I* (number of infected hosts) and, by extension, ${\rho }_{ij}$ is the probability of traveling to Rome from country *j* during week *i* while *I*_*ij*_ is the number of infected human hosts recorded in country *j* at week *i*. Therefore, the expected number of infected travelers to Rome from country *j* during week *i* (*N*_*ij*_) and its credible intervals can be estimated by simulating draws from a binomial distribution (details available in Technical Appendixes, section 4).

Weekly traveler counts by country of origin at Rome Fiumicino international airport were obtained from Banca d’Italia^27^ for two categories: visitors and residents. Visitors are travelers arriving directly from their resident country to Rome and staying at least one night in Rome. Residents are (returning) travelers whose resident address is in Rome municipality. In case of travelers returning from multiple destinations, we considered the last country visited as country of origin. For visitors, we estimated the weekly probability of traveling to Rome by dividing the weekly number of visitors from each country by their country population size. A portion of infected host may be not infectious at the moment of traveling, so the probability of travel was corrected by multiplying it by the length of infectious period (in days) to seven (days) fraction. For residents, we set the weekly probability of returning to Rome (their home residence) equal to 1.

Annual incident cases for DENV, CHIKV, ZIKV for 2013, 2014 and 2015 were obtained from available WHO reports (Table S1) and corrected for under-reporting rates for each virus: DENV^28^, CHIKV^29^, ZIKV^30^ (See Technical Appendixes, section 4).

The temporal pattern (by epidemiological week) of infected cases in endemic countries were extracted from the 2013–2015 annual series of DENV for a subset of countries (herein after “reference countries”) from which this information was publicly available either from national websites reporting official data (e.g. the Health Ministry), from the regional offices of WHO or from scientific publications (see Table S2 in Technical Appendixes for consulted websites). The temporal pattern of DENV cases was taken as reference also for CHIKV and ZIKV. The temporal pattern of DENV in reference countries was also used to distribute proportionally the yearly number of cases in similar endemic countries where no temporal pattern were available (See Technical Appendixes, section 4).

Finally, to assess the impact of future potential outbreak, we performed a scenario analysis by increasing either two- or ten fold the number of incident cases in those countries with both a highest air travelers flow with Rome and largest number of incident cases in 2012–2015 (i.e. Brazil, Dominique Republic, India, Mexico, French Polynesia, Thailand).

The model and all statistical analysis were carried out with the R software^31^.

## Results

In order to assess the risk of exotic arbovirus outbreak in Rome under different scenarios of human biting rates (HBR), we first predicted the weekly risk of autochthonous transmission and the probability of introduction of an infected traveler from endemic countries. We then used these estimates to calculate the overall probability of outbreak under different scenarios of incident cases reported worldwide.

### Autochthonous transmission (*R*_0_)

**Figure 1:**
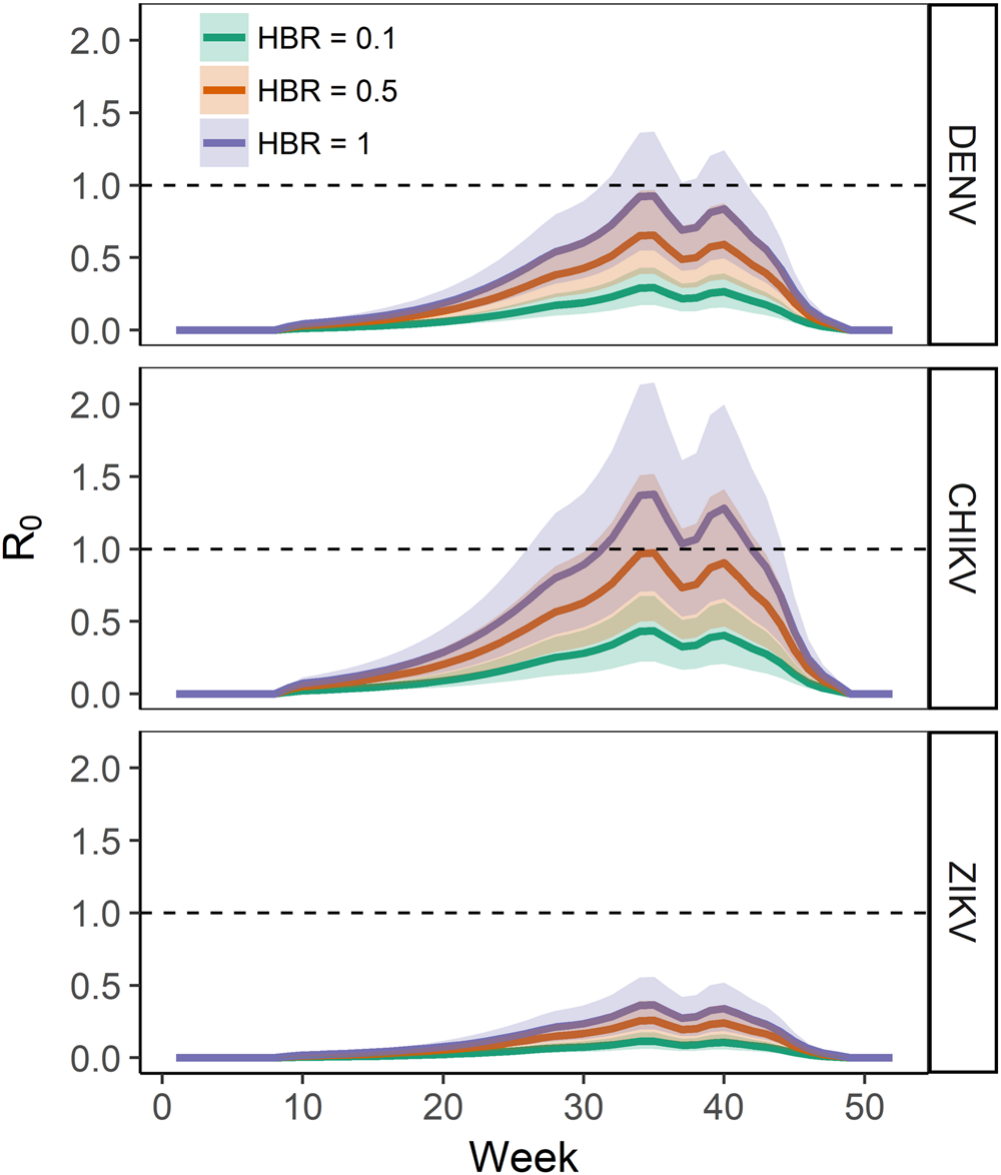
Estimated temporal patterns of *R*_0_ for Dengue (DENV), Chikungunya (CHIKV) and Zika (ZIKV) viruses under three different scenarios of daily human biting rates (HBR = 0.1 green lines; HBR = 0.5 red lines; HBR = 1 blue lines). On the x-axis the week of the year, on the y-axis the *R*_0_ value. Solid lines represent the mean values, shaded area represent the 95% credible interval. The horizontal dashed line indicates the threshold *R*_0_ = 1.

We estimated the risk of arbovirus autochthonous transmission under three scenarios corresponding to 10% (HBR = 0.1), 50% (HBR = 0.5) and 100% (HBR = 1) of human hosts bitten daily, under the hypothesis of homogeneous mixing (Technical Appendixes, section 3) and described the temporal dynamics of the risk based on the actual *Ae*. *albopictus* seasonal dynamics in Rome (Fig. S1). Our model predicted the possibility of secondary transmission for DENV and CHIKV (i.e. upper limit of the credible interval of the estimated mean of *R*_0_ > 1) when HBR ≥ 1 or ≥0.5, respectively (Fig. 1). No secondary transmission was predicted for ZIKV (Fig. 1). Secondary transmission was estimated to be possible from August to first half of October (weeks 31–42) for DENV at HBR = 1 and CHIKV at HBR = 0.5, and from second half of June to second half of October (weeks 26–44) for CHIKV at HBR = 1.

### Probability of arrival of infected travellers

**Figure 2:**
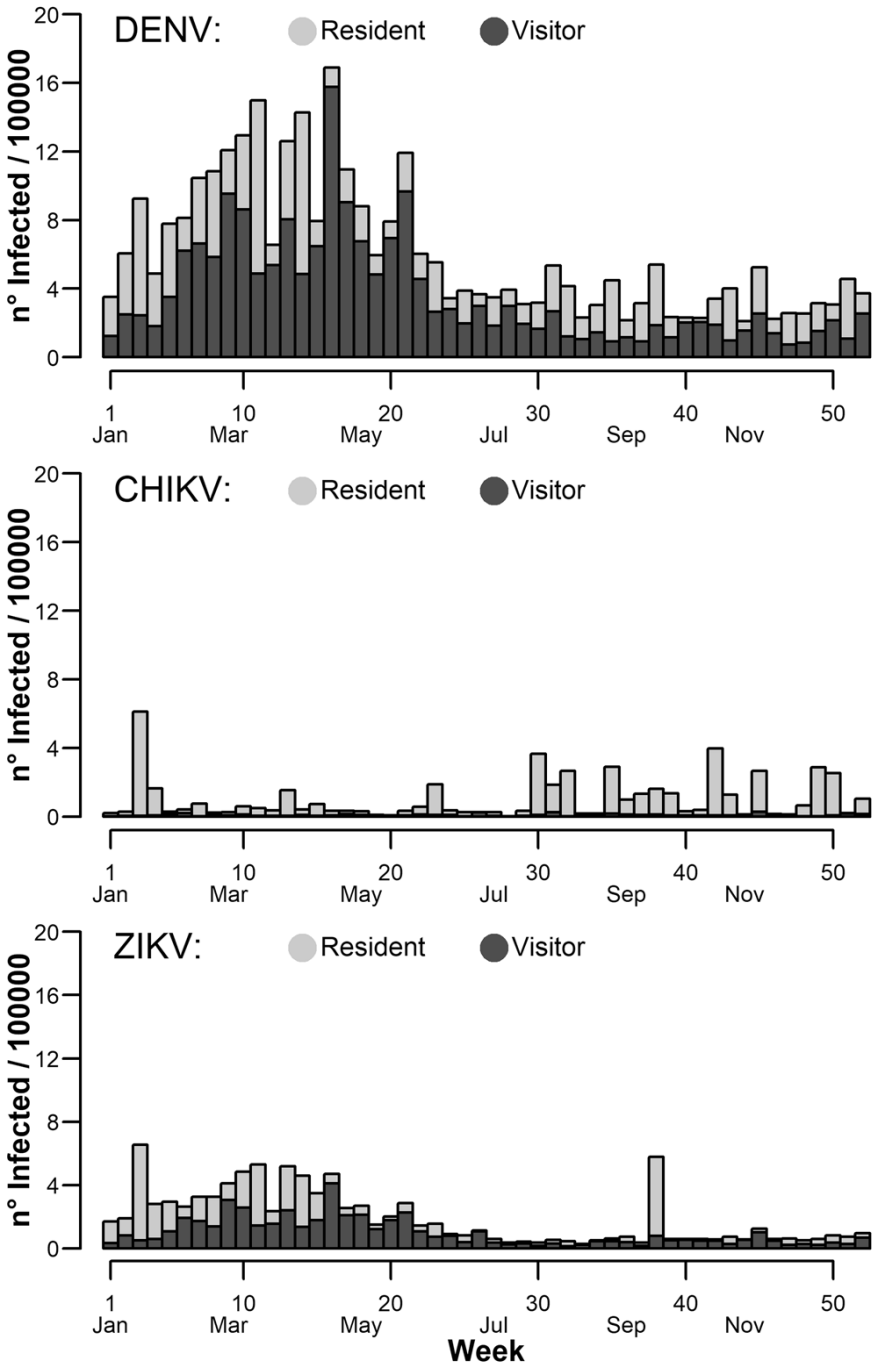
Estimated temporal pattern of worldwide incident cases for Dengue (DENV), Chikungunya (CHIKV) and Zika (ZIKV) viruses in travelers landing at Rome international airport. On the x-axis the week of the year, on the y-axis the number of infected per 100,000 travellers. Light grey bars represent the average number of resident travelers, dark grey bars represent the average number of foreign visitor travellers.

We estimated the weekly proportion of infected travelers arriving through the international airport of Rome Fiumicino from arbovirus endemic countries and sojourning for at least one night in Rome based on the annual means of visitors (7,256,831/year) and returning residents (2,214,806/year) in the period 2013–2015 (Fig. 2; Fig. S3) and on annual incident cases worldwide (Table S1 and S4). Arrivals of DENV infected travelers were predicted throughout the year (mean: 6.1; CI: 4.9–7.4/100,000 travelers) with higher proportions (>10/100,000 travelers/week) from the first half of February to end of May (weeks 7–21) and a peak of 16.9/100,000 travelers in the first half of April (week 16). The temporal pattern of CHIKV infected travelers (mean: 1, CI: 0.5–1.6/100,000 travelers) was less regular and with lower peaks (>3/100,000 travelers/week) predicted at weeks 3, 30, 42 and the highest peak (6 infected travelers/100,000 travelers/week) in second half of January (week 3). Lower proportions of ZIKV infected travelers (>2 per 100,000 travelers/week) were estimated from January to early May (weeks 1–18), while afterwards the proportion was estimated to be negligible with the exception of week 38 (second half of September), when the estimated proportion of infected reached 5.8/100,000 travelers. Resident returning travelers were the main introduction pathway for CHIKV, while visitor travelers were estimated in similar proportion for DENV and ZIKV (Fig. 2).

Regarding the different WHO regions, PAHO (Pan American Health Organization) was estimated to be the main source of infected travelers during the whole year for all viruses, while SEARO (South-East Asia), EMRO (Eastern Mediterranean) and WPRO (Western Pacific) regions had a smaller and scattered risk of infection (Fig. S3) and a lower traveler flow (Fig. S4).

**Figure 3:**
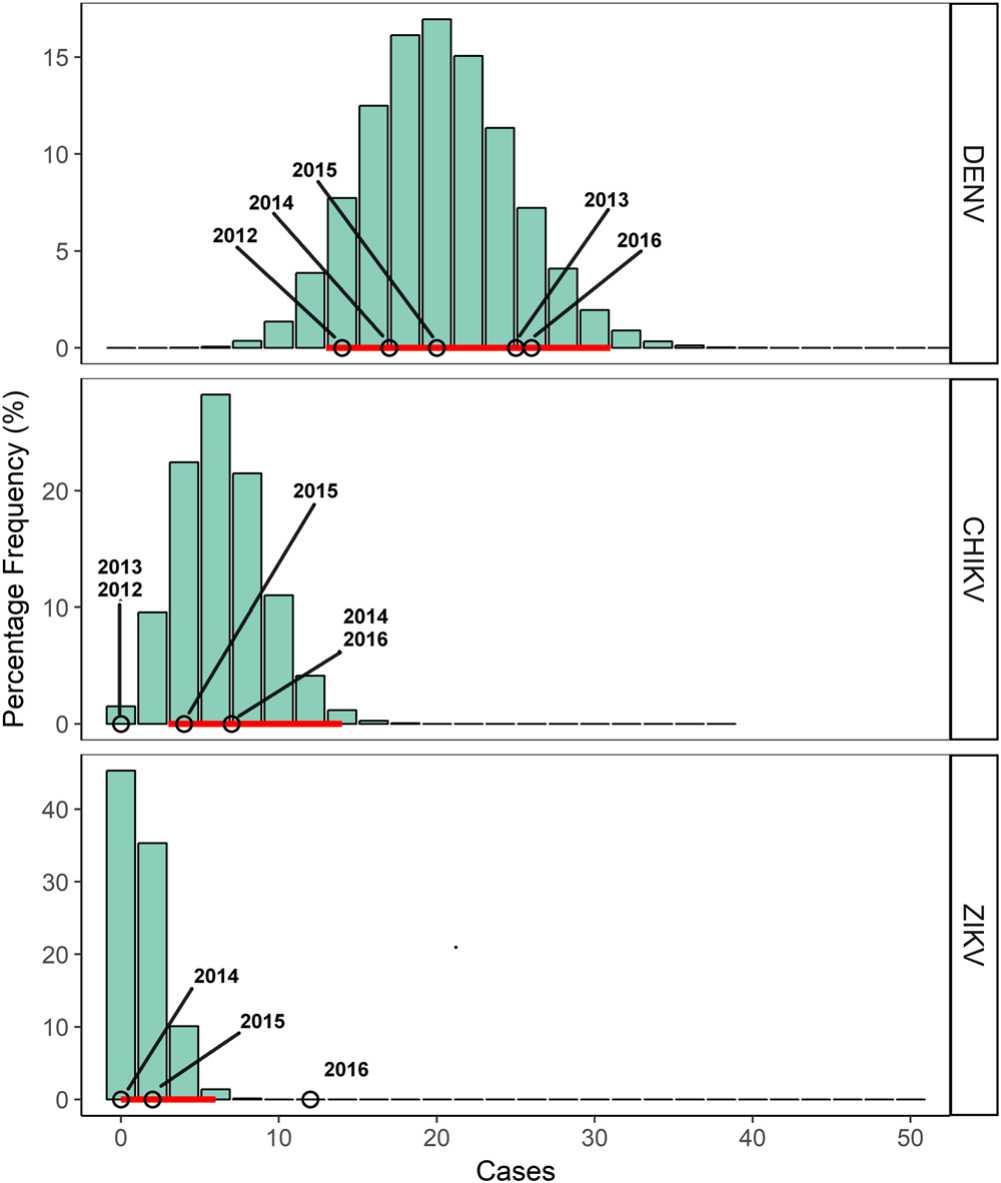
Frequency distributions (%) of predicted cumulative numbers of reported incoming travelers infected by Dengue (DENV), Chikungunya (CHIKV) and Zika (ZIKV) viruses. The histograms are the frequencies (%) of reported incoming infected travelers obtained from model runs while the horizontal red line is the 95% credible interval of predicted cases. Circles are the officially notified cases in Lazio region^33,34^.

The mode of the expected number of yearly cumulative infected travelers to Rome was 98 for DENV (95% Credible Interval 79–118), 14 for CHIKV (95% Credible Interval 8–22) and 26 for ZIKV (95% Credible Interval 16–36) (Fig. 3). The number of notified cases in Lazio region falls within distributions of expected cases (Fig. 3).

### Probability of outbreak in Rome

**Figure 4:**
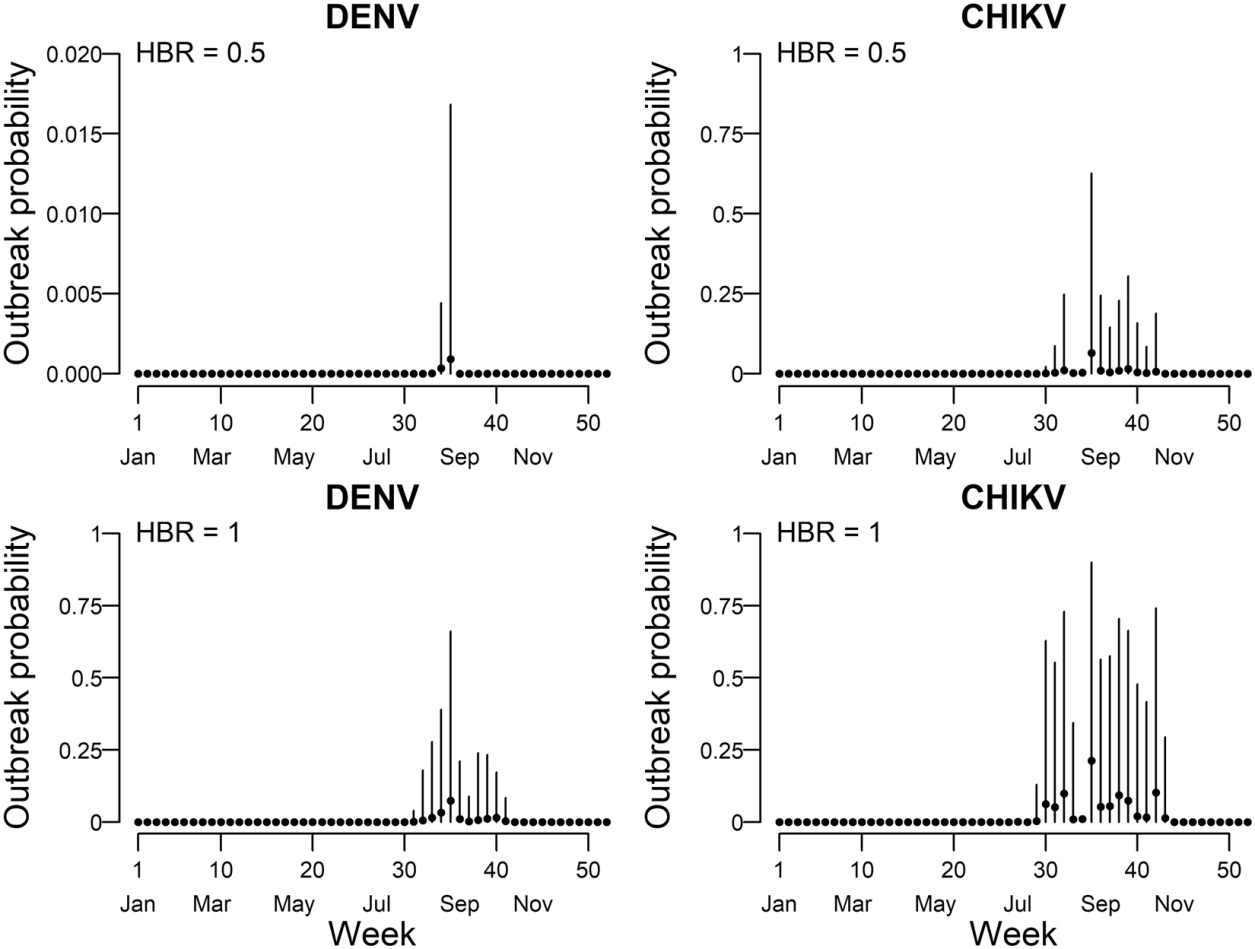
Weekly mean estimates and 95% credible interval of predicted probability of Dengue (DENV) and Chikungunya (CHIKV) virus outbreaks under two different scenarios of daily human biting rates (HBR = 0.5 upper panels; HBR = 1 lower panels). Dots represent the mean outbreak probability, the vertical lines represent their 95% credible intervals.

We estimated the mean probability of outbreak in Rome adopting measured flow of resident and visitor travelers to Rome and global number of incident cases in the 2013–2015 period (Fig. 4).

The yearly mean probability of outbreak was null for ZIKV at all levels of HBR and for DENV and CHIKV at HBR < 0.5 (data not shown) and negligible for DENV at HBR = 0.5 (Fig. 4). The mean outbreak probability was <0.1 at HBR = 1 for DENV (Fig. 4) and at HBR = 0.5 for CHIKV (Fig. 4), and increased up to 0.21 at HBR = 1 for CHIKV (week 35) (Fig. 4). The resulting annual cumulative probability of outbreak at HBR = 0.5 was 0.001 for DENV and 0.11 for CHIKV while it was 0.13 for DENV and 0.46 for CHIKV at HBR = 1.

**Figure 5:**
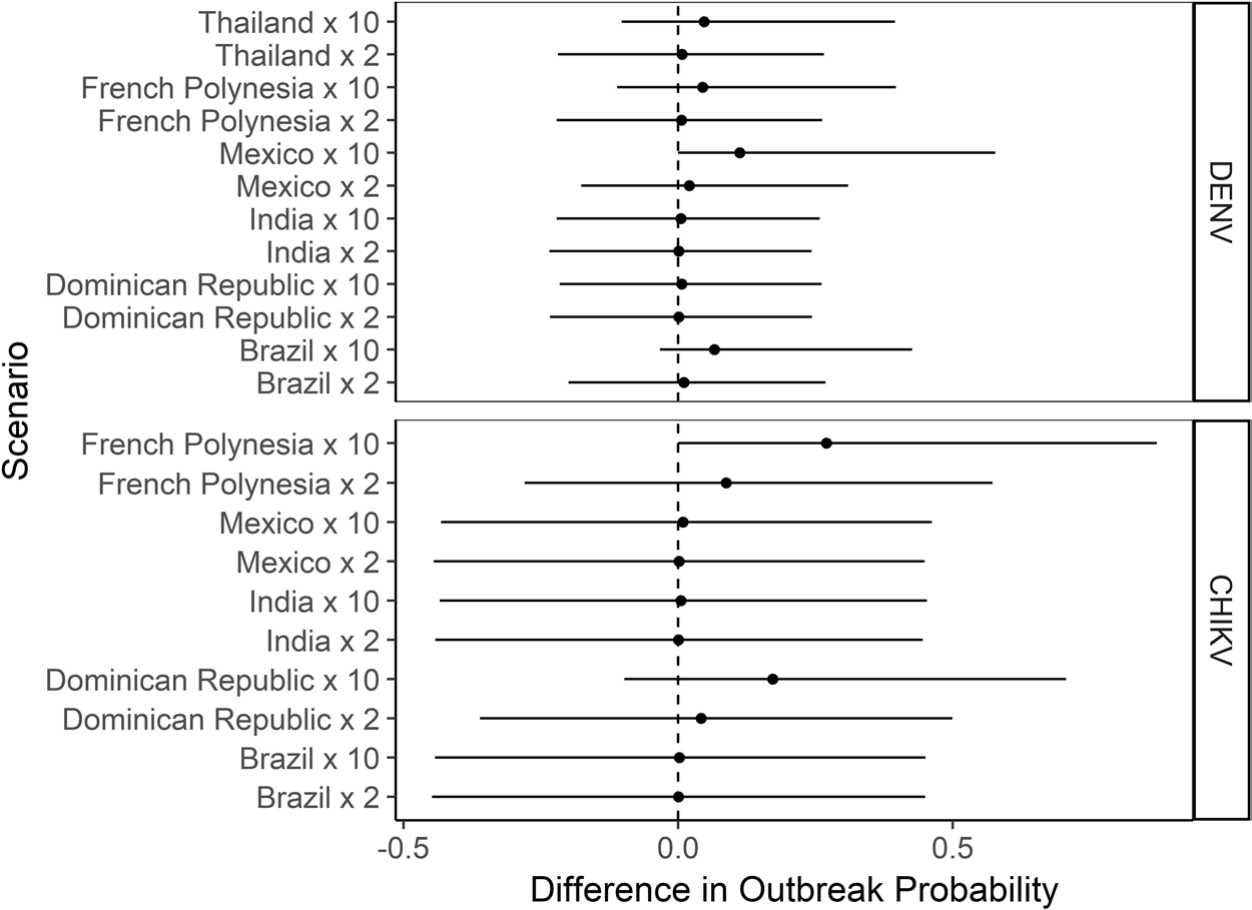
Yearly cumulative outbreak probability for Dengue (DENV) and Chikungunya (CHIKV) viruses in Rome in the case of HBR = 1. Differences in outbreak probability are shown considering 2012–2015 incident cases worldwide as baseline and comparing them with increasing epidemics (x2, x10) in selected countries. Dots represent the mean difference between baseline and scenario; solid horizontal lines represent the 95% credible interval of the difference distributions. The dashed vertical line indicates no difference.

Keeping the baseline flow of travelers from selected countries to Rome stable at HBR = 1, the model predicted that the cumulative yearly probability does not significantly increase with increasing incident cases (Fig. 5), with the exceptions of major DENV epidemics (x10 increase of incident cases) in Mexico and CHIKV in French Polynesia (Fig. 5).

## Discussion

Our modeling exercise based on available data on air passenger flow, incident cases worldwide and on actual entomological field data showed that the outbreak risk in Rome is higher for CHIKV than for DENV and null for ZIKV. The probability of CHIKV-outbreak was estimated to be very low and restricted in time at the HBR = 0.5 scenario, and much higher and extended from mid-June to October at HBR = 1. During the 2017-CHIKV outbreak in Rome, the estimated vector to host ratio at the time of the earliest notified case was 0.4–2.6^26^, which corresponds to a daily HBR between 0.02 and 0.23 (see Technical Appendixes 3). However, *Ae*. *albopictus* abundance estimates in Rome^25^ suggest that it is likely that the majority of the population experiences even lower daily HBR for most of the summer season. It is important to note that values of HBR > 1 may be expected during the peak of the mosquito reproductive season in specific hot-spots areas within the city (e.g. in small urban green areas^25^ or in the cemetery^32^), as shown by the very high number of landing *Ae*. *albopictus* females (up to 40) collected through human land catch (HLC) by a single volunteer in 15′^13^.

Overall, the model estimates a very low proportion of infected travelers arriving in Rome each year, i.e. 6.1 (CI = 4.9 −7.4) every 100,000 travelers for DENV, 1.9 (1.2–2.7) for ZIKV and 1 (0.5–1.6) for CHIKV. These estimates match the number of notified cases^33–35^ and improves an early attempt to estimate the flux of infected travelers based on airline tickets and modeled DENV global incident cases^36^. Notably, the estimated figure of infected travelers is mostly made up of returning residents, in agreement with data of the National Surveillance System according to which nearly 80% of CHIKV and DENV imported cases are returning travelers residing in Italy^37^.

Importantly, the period of August is predicted to be conductive for DENV and CHIKV transmission, but this time window can be extended from June to October in hot-spots of *Ae*. *albopictus* abundance. Since our model predicts for this period the arrival of a maximum of 9 infected visitors, it is unlikely - although possible - that an *Ae*. *albopictus* female bites one of those infected individuals (out of a total of approximately 3,000,000 of inhabitants) and initiates the outbreak. It should be noted that the model assumes homogeneity in the spatial distribution of mosquitoes and hosts while both have (possibly different) aggregate distributions. For example, it is likely that most visitors distribute in the central area of the city where most touristic attractions are located (e.g. Vatican City State). On the other hand, vector abundance is higher in residential areas where small houses with garden are located and where the vector to host ratio is critically high. This is consistent with the higher proportion of as CHIKV autochthonous cases registered in Anzio (a small town about 60 km South of Rome) as opposed to Rome during the 2017 CHIKV outbreak^26^. The higher outbreak risk for CHIKV compared to DENV is also the result of the higher competence of *Ae*. *albopictus* for the former virus^38^. Notably, the outbreak probability of DENV and CHIKV slightly changed when we simulated epidemics of increased intensity in 5 countries that have a large exchange of travellers with Rome. This is due to a combination of relatively low incidence proportion of CHIKV and DENV in the endemic countries, a short viremic period and the low fractions of travellers to Rome compared to the total population.

As in all modelling exercises, our model has some limitations. First, WHO estimates of country specific annual arbovirus cases are known to be prone to errors (see^39^). We assessed the temporal pattern of incident 2012–2015 DENV cases from reports available on official websites of national public health organisations (e.g. the national health ministry) or WHO regional offices. Therefore, the reliability of the data may differ according to the country specific technical sophistication of case assessment and reporting procedure. Second, we used DENV incident case temporal pattern to infer seasonal transmission dynamics and applied the same temporal pattern to CHIKV and ZIKV simulations and to all countries in the same WHO sub-region. Third, estimates for key epidemiological parameters, such as *Ae*. *albopictus* competence for ZIKV, are based on a limited number of experiments. This implies that model parametrization ought to be updated when more recent and reliable estimates will be available. Fourth, outbreak probability is computed without considering possible reactive interventions carried out by public health authorities after the detection of each case in order to reduce the transmission risk.

Finally, although a formal sensitivity analysis of model parameters was not attempted, we inferred the uncertainty of model output by simulation. Each parameter was resampled 100,000 times from parameter-specific sampling distributions as done by others^40,41^, resulting in an ensemble of possible scenarios rather than a parameter dependent one. Therefore, by calculating the most reliable interval of outbreak probability over the 100,000 simulations we implicitly included the uncertainty in the model output generated by model parameters.

## Conclusion

Our study provides the first quantitative estimation of DENV, CHIKV and ZIKV outbreak risk in south European metropolitan area and allows to predict several patterns of the potential outbreak: (i) which arbovirus is most likely to cause and outbreak, (ii) in which period of the year the probability of secondary transmission is highest, (iii) which type of travelers and origin of flights are more likely to cause virus importation, and (iv) the relative importance of an increase in vector density or in the global number of incident cases. The approach proposed can be easily adapted to other cities for which data on the temporal dynamics of travelers and seasonal dynamic of *Ae*. *albopictus* are available and serve as guide for public health officers in charge of preventing arbovirus outbreaks.

Given the latest 2017 CHIKV outbreak in the town of Anzio nearby Rome with limited propagation of the outbreak to Rome iteself^1^, next research should focus on factors influencing local transmission at the spatial scale relevant for the host-vector contact. Since both the vectors and the human population exhibit an aggregate spatial distribution, a more precise risk model should tackle the relevant spatial scale where those distributions do overlap with the probability of arrival of an infected traveler.

## Electronic supplementary material

Supplementary Information

## Data Availability

The data used as model inputs are third party data freely available.
