# Supplementary material for: Estimating the risk of Dengue, Chikungunya and Zika outbreaks in a large European city
Source: Sci Rep. 2018 Nov 6;8:16435. doi: 10.1038/s41598-018-34664-5 (PMC6219586; doi:10.1038/s41598-018-34664-5)
Supplement: Supplementary file 1 — Supplementary Information [file 41598_2018_34664_MOESM1_ESM.docx]

**SUPPLEMENTARY MATERIAL**

**Estimating the risk of Dengue, Chikungunya and Zika outbreaks in a large European city**

**Angelo Solimini^1*^, Mattia Manica^1,2^, Roberto Rosà^2^, Alessandra della Torre^1^ and Beniamino Caputo^1^.**

1. Department of Public Health and Infectious Diseases, Universita’ La Sapienza, Rome, Italy. *Corresponding author: Angelo G. Solimini (angelo.solimini@uniroma1.it)

2. Department of Biodiversity and Molecular Ecology, Research and Innovation Centre, Fondazione Edmund Mach, San Michele all’Adige (TN), Italy

**Technical Appendixes**

***1. Derivation of Probabilistic model***

If N is a discrete random variable (on the non-negative integers) and X_1_,…,X_N_ are discrete independent identically distributed random variable (on the non-negative integers), then the sum $S_{N}=X_{1}+\ldots+X_{N}$ has the probability generating function $G_{S_{N}}\left( s \right)=G_{N}\left( G_{X}\left( s \right) \right)$. In our approach, N is the number of secondary infection generated in the vector by a single infected host while the random variable X describes the number secondary infections generated in host after an infectious bite from a vector. Classical theory of vector-host models (Ross model) assume that the hosts have a constant rate of recovery while the infected vectors have a constant mortality rate. It follows that the duration of infection is exponentially distributed for both host and vector. Moreover, in this context it has been demonstrated (Lloyd et al 2007, Diekmann & Heesterbeek 2000) that if secondary infections arise independently and at a constant rate then the distributions of secondary infections from host to vector and also from vector to host follow a geometric distribution. Given that there are two classes of individuals (vector and host), the generating function can be expressed as $G_{X,i}\left( s_{H},s_{V} \right)=\sum_{z_{H},z_{V}} s_{H}^{z_{H}}s_{V}^{z_{V}}P\left( X_{iH}=z_{H},X_{iV}=z_{V} \right)$ where *i=H,V* and $X_{iH}$is the random variable describing the number of secondary infection of class i from an individual of class *H.* Moreover, it follows from the assumption that there is no direct transmission between individuals of the same class that $P\left( X_{HH}=z_{H},X_{HV}=z_{V} \right)=P\left( X_{HV}=z_{V} \right)$ only if $z_{H}=0$ otherwise $P\left( X_{HH}=z_{H},X_{HV}=z_{V} \right)=0$. Now, it is possible to compute the composite probability generating function for the complete process and from it compute the extinction probability of infection arising from a single infected host as a function of $R_{0}^{VH}$ and $R_{0}^{HV}$that are the mean of the geometric distribution of secondary infections from vector to host and host to vector, respectively. $R_{0}^{VH}$ and $R_{0}^{HV}$ define the typical two-stage process of transmission for mosquito-borne arboviruses involving, for example, at first the transmission from an infected host to a suitable vector and the following transmission from the infected vector to a susceptible host. The basic reproduction number (*R*_0_) can be defined as the product between the numbers of infectious mosquitoes generated from an infectious human (*R_0_^HV^*) and the number of infectious humans generated by the infectious mosquitoes surviving the extrinsic incubation period (*R_0_^VH^*). Mathematical modelling has demonstrated that both these quantities can be computed as a function of parameters (Table 1) describing the pathogen transmission or the mosquito life: $R_{0}^{HV}=\frac{k\chi_{V}}{\gamma}\frac{V}{H}\frac{\omega_{V}}{\omega_{V}+m}$ and $R_{0}^{VH}=\frac{k\chi_{H}}{m}$ (Smith et al., 2012, Poletti et al. 2010). When R_0_ < 1 (epidemic threshold), the probability of observing sustained arbovirus transmission after importation of a single case is negligible. When R_0_ > 1, the extinction probability is then equal to: $\frac{R_{0}^{VH}+1}{R_{0}^{HV}\left( R_{0}^{HV}+1 \right)}$ and hence the probability of outbreak could be defined as$p=1-\frac{R_{0}^{VH}+1}{R_{0}^{VH}(R_{0}^{HV}+1)}$ as demonstrated by (Lloyd et al 2007).

On the other hand, the number of infected human hosts that travel to Rome follows a binomial distribution of parameter $\rho$ (probability of arriving) and *I* (number of infected host) and by extension $\rho_{ij}$ is the probability traveling to Rome from country *j* during week *i* and *I_ij_* is the number of infected human host in country *j* in week *i*. Therefore, the expected number of infected human host traveling to Rome from country *j* during week *i* (*N_ij_*) and its credible intervals could be estimated by simulating draws from the defined binomial distribution. Its generating function is $G_{N}\left( s \right)=\left( 1-\rho+\rho s \right)^{I}$. Then, for each infected host the probability of not observing an outbreak could be defined as the sum of the probabilities of two events: the probability of the infected host not arriving in Rome ($1-\rho$ and the probability that he/she arrives but there won’t be an outbreak $\left( \rho\frac{R_{0}^{VH}+1}{R_{0}^{HV}\left( R_{0}^{HV}+1 \right)} \right)$. If infected hosts are assumed to be independent then the cumulative probability of never observing a major outbreak could be written as $\prod_{j} \prod_{i} \left( 1-\rho_{ij}+\rho_{ij}\frac{R_{0, i}^{VH}+1}{R_{0, i}^{HV}\left( R_{0, i}^{HV}+1 \right)} \right)^{I_{ij}}$ and subsequently the probability of observing at least one could be computed as $1-\prod_{j} \prod_{i} \left( 1-\rho_{ij}+\rho_{ij}\frac{R_{0, i}^{VH}+1}{R_{0, i}^{HV}\left( R_{0,i}^{HV}+1 \right)} \right)^{I_{ij}}$. Moreover, one can allow for different $\rho_{ij}$ (probability of travelling to Rome) based on the knowledge of the travelers being resident or visitors. Therefore, defined *t* as an index indicating if the traveler is resident or visitor the equation is $1-\prod_{j} \prod_{i} \prod_{t} \left( 1-\rho_{ijt}+\rho_{ijt}\frac{R_{0,i}^{VH}+1}{R_{0,i}^{HV}\left( R_{0,i}^{HV}+1 \right)} \right)^{I_{ijt}}$

***2. Derivation of Temperature estimate***

A Generalized Additive Model (GAM) has been fitted to the weekly series of mean temperature recorded in Rome between 2003-2014 in order to estimate an overall yearly temperature pattern.

Data were collected by the Hydrographic Service of Regione Lazio and disseminated through the hydrographic annals (http://www.idrografico.roma.it/annali). The records of the meteorological sampling station “Roma Sud” were used. The generalized additive model estimated the parameter of the smoothing function of the week of year that best fitted the weekly series of mean temperature.

Then the weekly temperature estimates to be used in each simulation run were sampled from a normal distribution having as mean the corresponding weekly temperature estimated from the GAM and standard deviation equal to the square root of the variance of the predicted mean temperature.

***3. Derivation of Biting Scenario***

The human biting rate (HBR) has been defined has the product between the mosquito biting rate (ie how often a single mosquito bites) and the vector to host ratio (i.e. the number of mosquito per person). The HBR indicates the number of bites an individual should expect per unit of time (day). The values for the human biting rate (0.1, 0.5 and 1) were chosen based on previous results of *i)* a mark-release experiments conducted in a spatially defined area of 10.6 Ha in central Rome during the summer of 2008 (Marini et al, 2010), *ii)* previous field experiment in Rome province (Manica et al 2016) and *iii)* recent model estimates subsequent to a local Chikungunya outbreak (Manica et al 2017).

1. In the mark-release experiments, *Aedes albopictus* were released three time: in August, September and October. Results of the study showed a median (over different estimating methods) vector density per hectare of 52, 99, 653.5 depending on the release. If one assumes a spatially uniform distribution of *Ae. albopictus* and human host over the study area (, then it is possible to compute the vector to host ratio by dividing the estimated vector density by the human host density in the study area, which is known to be 122.7 person per hectare (Municipality II, Rome metropolitan city). In order to compute the bites per human host we used a biting rate of 0.09 (Poletti et al 2010, Guzzetta et al 2016), thus resulting in a human biting rate of 0.04, 0.07 and 0.48 respectively.
2. In the 2012 field experiments 4 Sticky traps (Facchinelli 2007) designed to capture ovidepositioning adult females was placed within each one of 18 sites along a 70 km transect encompassing the metropolitan city of Rome. 9 sites where located within the city limits. By the mark-release-recapture study in point *i)* one can roughly estimate the mean capture rate (~ 0.0008) by dividing the total recaptured by the total released. Then we considered the mean number of captured females during the week of seasonal peak activity and divided it by the capture rate to obtain an estimate of flying females. The latter estimated was used to compute the HBR together with the capture rate (0.09) and the estimated number of resident citizens in each site (obtained by ISTAT, Italian census 2011). These computations resulted in an HBR in the range of 0.02 – 0.23.
3. Recently a stochastic population model (Manica et al 2017) was developed to describe the 2017 Italian Chikungunya outbreak. The model was calibrated on the same data on point *ii)* but then fitted using 2017 mean temperature in Rome. The model estimates of vector to host ratio within the study sites (~28 hectares each) at the time of the erlieast notified case (June 26^th^, 2017, WHO report at 6^th^ October 2017) were 0.4 - 2.6 in the surveyed urban sites. Multiplying that number by the biting rate resulted in an HBR between 0.036 and 0.234. Considering mosquito to host ratio during the week of seasonal peak activity showed that the HBR could be even higher than 1 in some heavily infested sites.

Therefore, we hypothesized three scenarios for the HBR ranging from 0.1 to 1.

***4. Estimates of the number of infected travellers***

Annual incident cases for DENV, CHIKV, ZIKV for 2013, 2014 and 2015 were obtained from available WHO reports (table S1). To correct for under-reporting, the total number of cases was multiplied by the inverse of the reporting rate for each virus (DENV: Shepard 2014, CHIKV: Moro 2010, ZIKV: Guzzetta 2017). The corrected annual number of cases was set as the mean parameter of a Poisson distribution from which we draw the number of infected in each simulation run.

To account for the temporal pattern in incident cases, the 2013-2015 annual series of DENV cases by epidemiological week were extracted for a subset of countries (herein after “reference countries”) from which this information was publicly available either from national websites reporting official data (e.g. the Health Ministry), from the regional offices of WHO or from scientific publications (see table S2 supplementary material for consulted websites). The temporal pattern of DENV cases was taken as reference also for CHIKV and ZIKV. For each reference country, to account for gaps and missing values in the case annual series, we fitted a generalized additive model to get the number of cases as a function of time. The fitted mean annual series of DENV cases of reference countries was then used to proportionally distribute the total cases along the year by epidemiological week (Figure S2). The temporal pattern for country with annual cumulative data, but with no information about the weekly number of cases, was assumed to be the same as the geographically closest reference country. Whenever the annual series of cases was not available for the whole 2013-2015 periods, most recent data available were used instead. In few cases, the annual series was reported by month and we equally distributed the monthly cases by the corresponding week. Only for African countries, for which only scattered information is available, the annual total of cases was uniformly distributed through the epidemiological weeks. Afterwards, the weekly number of infected hosts was adjusted by a proportion of cases reported in the following week to take into account delays due to symptom onsets (the proportion was computed as one minus the total length of latent and infectious period divided by seven). Finally, we assumed that half of the symptomatic would not travel, the symptomatic fraction being 24% for DENV (Shepard 2014), 82% for CHIKV (Moro 2010) and 20% for ZIKV (Zhang et al 2017). Therefore, not considering them as potential visitors travelers. However, the weekly number of cases arising in the resident travelers visiting an endemic country is yet to be estimated. Therefore, to have the weekly number of infected hosts that are potential resident travelers we sampled in each simulation run from a binomial distribution of mean equal to the fraction between the weekly number of infected hosts and the country population and size equal to the number of resident of Rome that have declared at least a one-night stay in that country during that week.

**Supplementary tables**

**Table S1**. List of websites or literature consulted for retrieving data on dengue temporal pattern. In case the information was reported in the form of a plot, a web based tool to extract data from plots was used (https://automeris.io/WebPlotDigitizer/).

|  |  |
| --- | --- |
| Africa | Baba et al. Repetitive dengue outbreaks in East Africa: A proposed phased mitigation approach may reduce its impact Rev. Med. Virol. 2016; 26: 183 –196. |
| Argentina | Ministerio de Salud; http://www.msal.gob.ar/salud |
| Bangladesh | Institute of Epidemiology, Disease Control and Research; http://www.iedcr.gov.bd/ |
| Brasil | Ministério da Saúde; http://combateaedes.saude.gov.br |
| Cambodia | WHO Western Pacific regional office; http://www.wpro.who.int/ |
| China | Lai et al. The changing epidemiology of dengue in China, 1990-2014: a descriptive analysis of 25 years of nationwide surveillance data. BMC Medicine 2015 13:100 |
| Colombia | Instituto Nacional de Salud; http://www.ins.gov.co |
| Costa Rica | Ministerio de Salud de Costa Rica; https://www.ministeriodesalud.go.cr |
| Dominica Republic | Ministerio de Salud Publica; http://digepisalud.gob.do |
| French Guyane | Santé publique France; http://www.invs.sante.fr |
| French Polynesia | Centre d’Hygiène et de Salubrité Publique; http://www.hygiene-publique.gov.pf |
| Guadalupe | Santé publique France; http://www.invs.sante.fr |
| India | Health & Family Welfare Department of Govt. of NCT of Delhi; http://idsp.nic.in |
| Indonesia | National Environment Agency; https://www.nea.gov.sg/ |
| Malaysia | WHO Western Pacific regional office; http://www.wpro.who.int/ |
| Maldives | Aishath et al. An evaluation of the surveillance system for dengue virus infections in Maldives. South-East Asia Journal of Public Health 2014, 3: 60-68 |
| Martinique | Santé publique France; http://www.invs.sante.fr |
| Mexico | Secretaría de Salud, Dirección General de Epidemiología; http://www.gob.mx |
| Panama | Ministerio de Salud; http://www.minsa.gob.pa/ |
| Philippines | WHO Western Pacific regional office; http://www.wpro.who.int |
| Puerto Rico | Departamento de Salud; http://www.salud.gov.pr |
| Saint Martin | Centre d’Hygiène et de Salubrité Publique; http://www.hygiene-publique.gov.pf |
| Singapore | WHO Western Pacific regional office; http://www.wpro.who.int |
| Sri Lanka | Epidemiology Unit, Ministry of Health; http://epid.gov.lk |
| Thailand | Department of Disease Control, Ministry of Public health; http://www.boe.moph.go.th |
| Venezuela | Observatorio Venezolano de la Salud; http:// www.ovsalud.org |
| Vietnam | WHO Western Pacific regional office; http://www.wpro.who.int |

**Table S2**. Cumulative number of cases (averages of 2012-2015) retrieved form WHO regional websites.

| **Country (or oversea territory)** | **Reference Country** | **DENV cases** | **CHIKV cases** | **ZIKV cases** | **Population (thousands)** |
| --- | --- | --- | --- | --- | --- |
| Anguilla | Collectivity of Saint Martin | 86 | - | 5 | 16 |
| Antigua e Barbuda | Collectivity of Saint Martin | 37 | 730 | 14 | 91 |
| Argentina | Argentina | 5499.7 | 122 | 815 | 41803 |
| Aruba | Collectivity of Saint Martin | 400 | 382 | 13 | 111 |
| Bahamas | Collectivity of Saint Martin | 146 | 92 | 15 | 383 |
| Bangladesh | Bangladesh | 1016.5 | - | - | 160996 |
| Barbados | Collectivity of Saint Martin | 2686 | 930.5 | 172.5 | 290 |
| Belize | Costa Rica | 3858 | 3 | 24.5 | 340 |
| Bermuda | Mexico | 1 | 0 | 0 | 70 |
| Bhutan | India | 906.5 | - | - | 775 |
| Bolivia | Colombia | 18388 | 71.5 | 113.5 | 10848 |
| Brazil | Brazil | 1236320.3 | 12857 | 129250.5 | 202362 |
| Cambodia | Cambodia | 11892.3 | - | - | 13395.7 |
| Canada | Mexico | 0 | 0 | 0 | 35525 |
| Cayman Islands | Collectivity of Saint Martin | 124 | 136 | 17 | 55 |
| Chile | Brazil | 34.5 | 0 | 0 | 17773 |
| China | China | 3884 | - | - | 1400000 |
| Collectivity of Saint Martin | Collectivity of Saint Martin | 1749.5 | 1068.5 | 365 | 40 |
| Colombia | Colombia | 109163.6 | 223585.5 | 45094.5 | 48930 |
| Costa Rica | Costa Rica | 26088.67 | 2527.5 | 844.5 | 4938 |
| Cuba | Dominican Republic | 1864.3 | - | 1.5 | 11266 |
| Curaçao | Collectivity of Saint Martin | 194 | 2673 | 161 | 148 |
| Dominica | Collectivity of Saint Martin | 7 | 1881.5 | 234.5 | 73 |
| Dominican Republic | Dominican Republic | 13267.3 | 269625 | 1657.5 | 10529 |
| East Timor | Indonesia | 640 | - | - | 1185 |
| Ecuador | Colombia | 23898 | 14738 | 1707.5 | 15983 |
| El Salvador | Costa Rica | 44163.7 | 67700 | 5859.5 | 6384 |
| French Guiana | French Guiana | 8721.5 | 44150 | 3841.5 | 255 |
| French Polynesia | French Polynesia | 1561.3 | 17360.5 | - | 268.77 |
| Granada | Collectivity of Saint Martin | 39 | 76 | 50 | 110 |
| Guadeloupe | Guadeloupe | 7051.5 | 7113.5 | 4104.5 | 468 |
| Guatemala | Costa Rica | 16569.7 | 14782 | 802 | 15860 |
| Guyana | Collectivity of Saint Martin | 863 | 3409.5 | 3 | 804 |
| Haiti | Dominican Republic | - | 64709 | 891 | 10461 |
| Honduras | Costa Rica | 42520.3 | 2676 | 10655 | 8261 |
| India | India | 46365.5 | 15767 | - | 1300000 |
| Indonesia | Indonesia | 66063.5 | - | - | 257564 |
| Jamaica | Collectivity of Saint Martin | 625.7 | 1697.5 | 371 | 2799 |
| Kenya | Africa | 346 | - | - | 38765 |
| Malaysia | Malaysia | 87776.3 | - | - | 29336.8 |
| Maldives | Maldives | 1362 | - | - | 364 |
| Martinique | Martinique | 4360 | 37028 | 13906 | 405 |
| Mexico | Mexico | 192011.3 | 5866 | 2591 | 123799 |
| Montserrat | Collectivity of Saint Martin | 17 | 63 | 0 | 5 |
| Mozambique | Africa | 176.5 | - | - | 25830 |
| Myanmar | Thailand | 5585.5 | - | - | 53897 |
| Nepal | India | 131 | - | - | 28514 |
| Nicaragua | Costa Rica | 53729.3 | 37189.5 | 1001 | 6169 |
| Pakistan | India | 3664 | - | - | 188925 |
| Panama | Panama | 4376 | 74.5 | 643.5 | 3926 |
| Paraguay | Brazil | 68423 | 1709.5 | 143.5 | 6918 |
| Peru' | Colombia | 24105.3 | 96 | 114 | 30769 |
| Philippines | Philippines | 160002.3 | - | - | 94013.2 |
| Puerto Rico | Puerto Rico | 9636.7 | 17759 | 19370.5 | 3688 |
| Saint Barthélemy | Collectivity of Saint Martin | 511 | 2970 | 50.5 | 9 |
| Saint Kitts and Nevis | Collectivity of Saint Martin | 75 | 655 | 16 | 52 |
| Saint Lucia | Collectivity of Saint Martin | 167 | 883 | 37 | 163 |
| Saint Vincent and the Grenadines | Collectivity of Saint Martin | - | 1393 | 19 | 103 |
| Singapore | Singapore | 17270 | 429 | - | 3771.7 |
| Somalia | Africa | 23 | - | - | 10790 |
| Sri Lanka | Sri Lanka | 36447.3 | - | - | 20715 |
| Sudan | Africa | 254 | - | - | 40240 |
| Suriname | Venezuela | 197 | 1210 | 1619 | 544 |
| Tanzania | Africa | 210 | - | - | 42484 |
| Thailand | Thailand | 63401.5 | - | - | 67959 |
| Trinidad and Tobago | Collectivity of Saint Martin | 5157 | 291 | 244 | 1344 |
| Turks and Caicos Islands | Collectivity of Saint Martin | 252 | 19 | 2 | 49 |
| United States of America | Mexico | 609 | 11 | 137 | 322583 |
| Uruguay | Brazil | 0 | 98 | 0 | 3419 |
| Venezuela | Venezuela | 69373 | 28026.5 | 16712 | 30851 |
| Vietnam | Vietnam | 63304 | - | - | 86025 |
| Virgin island | Collectivity of Saint Martin | - | - | 371.5 | 134 |

**Supplementary figures**


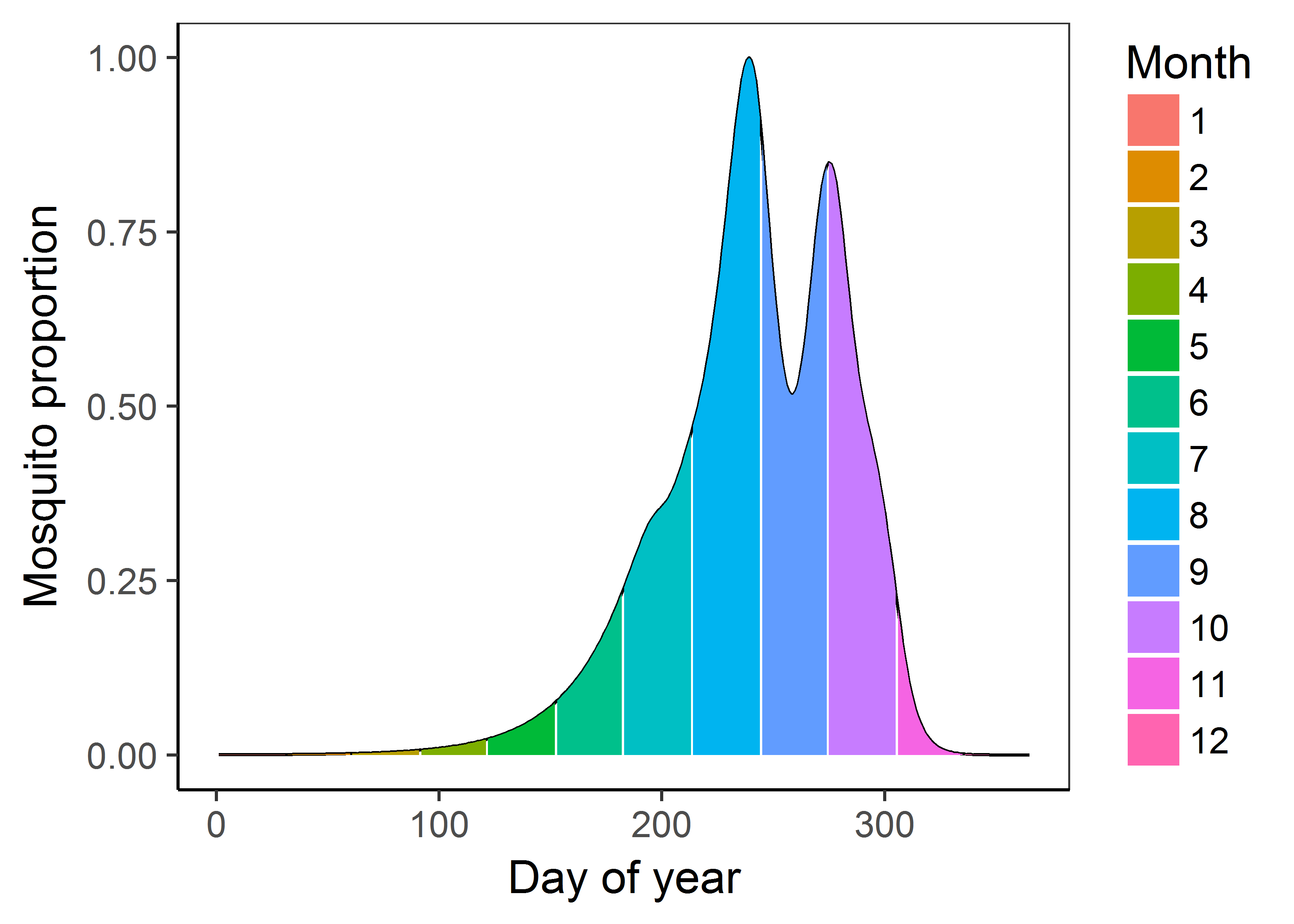


**Figure S1:** Temporal pattern of mosquito population as estimated using the Generalized Additive Mixed Model (GAMM) presented in Manica *et al.* 2016 applied to 2012 field mosquito data from Rome province. On the x-axis the day of the year, on the y-axis the proportion of mosquito abundance with respect to the estimated peak.


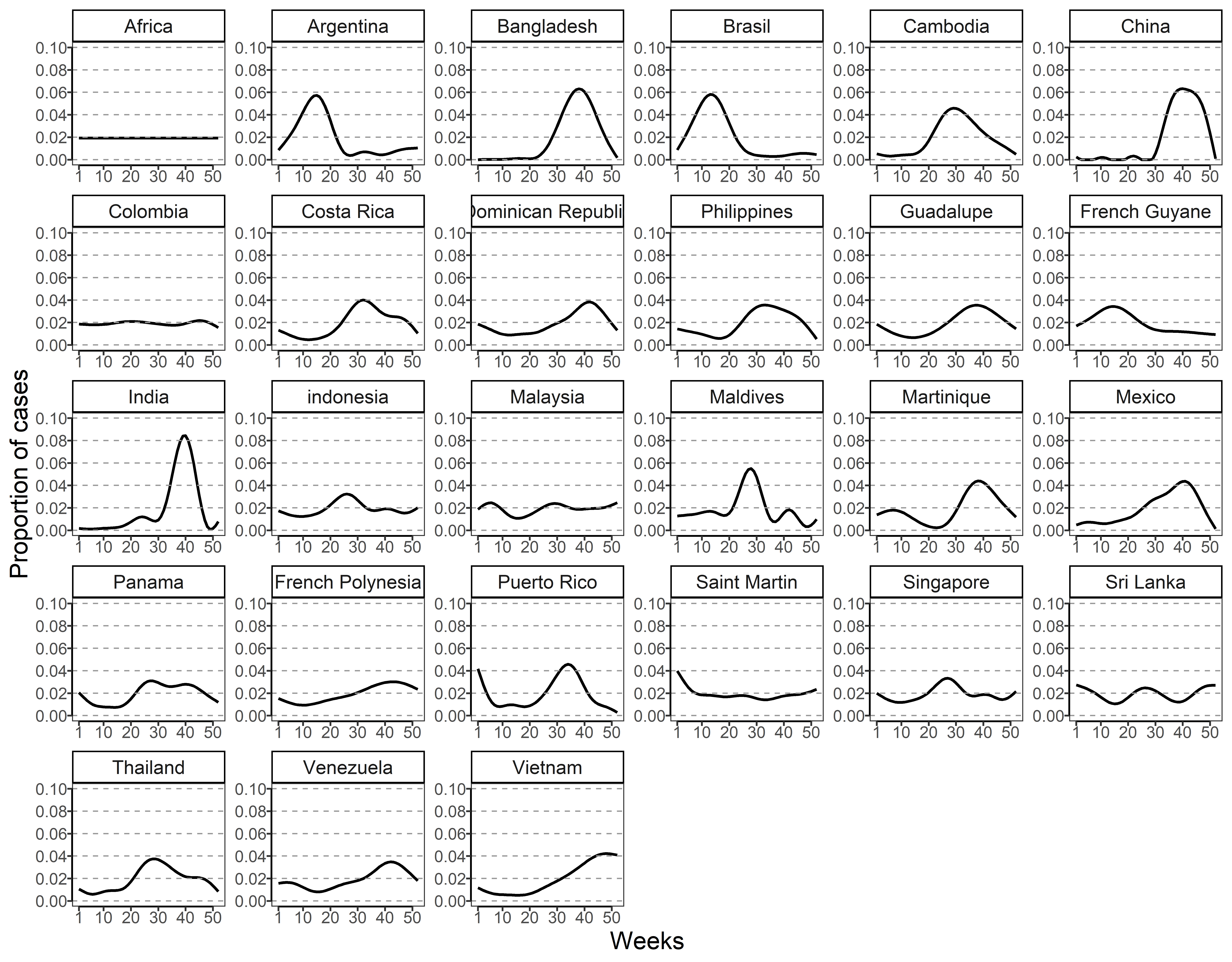


**Figure S2.** Smoothed proportional trend of notified cases in the reference countries. On the x-axis the weeks of the year. Each panel represents a reference country. On the y-axis the proportion of notified cases in the corresponding week compared to the yearly cases.


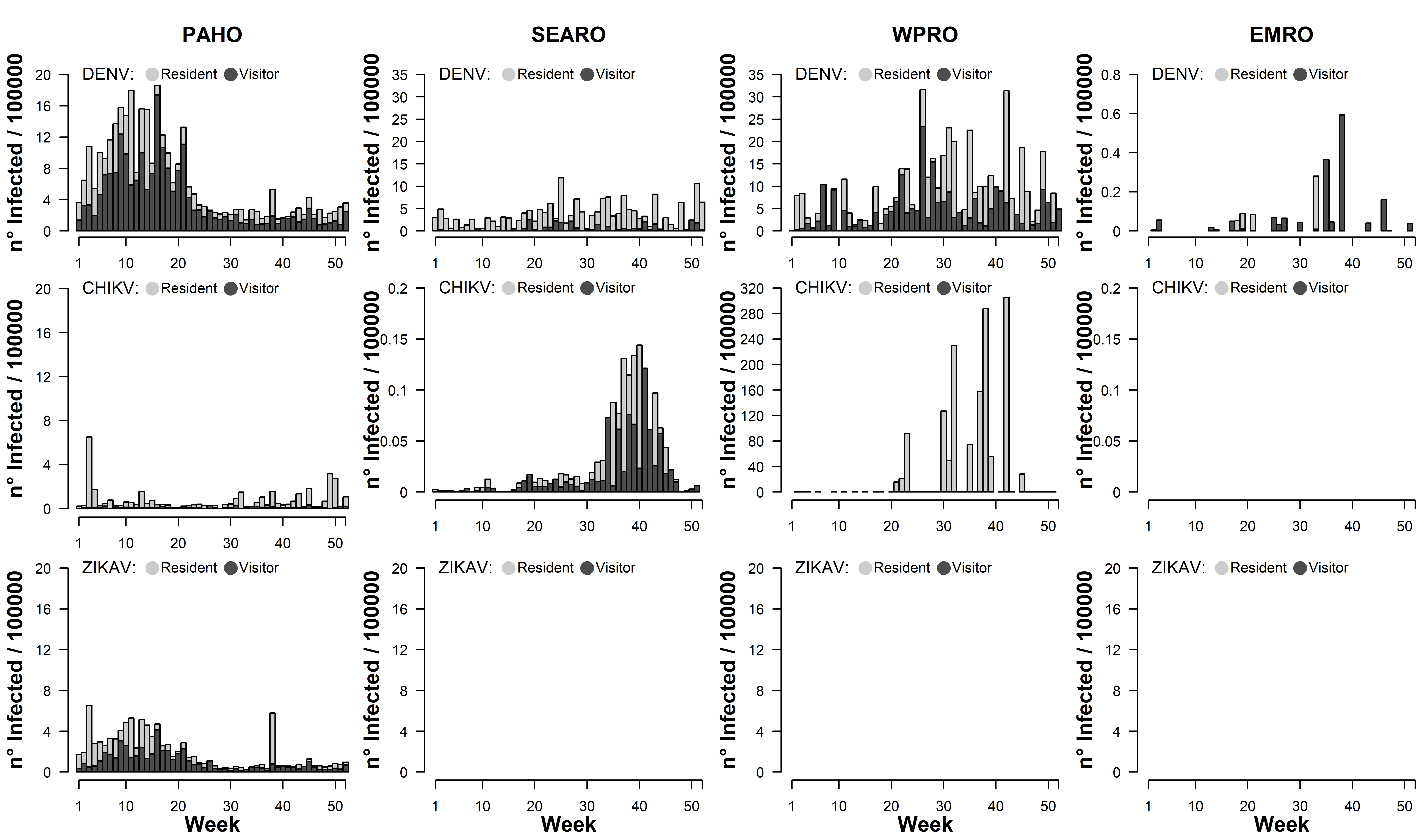


**Figure S3.** Estimated temporal pattern of incident cases of DENV, CHIKV and ZIKV in air travellers at Rome international airport grouped by WHO regions: PAHO (Pan American Health Organization) SEARO (South East Asia Regional Office), WPRO (Western Pacific Regional Office) and EMRO (Eastern Mediterranean Regional Office). On the x-axis the week number, on the y-axis the number of infected per 100,000 travellers. Light grey bars represent the average number of resident travellers, dark grey bars represent the average number of foreign visitor travellers.


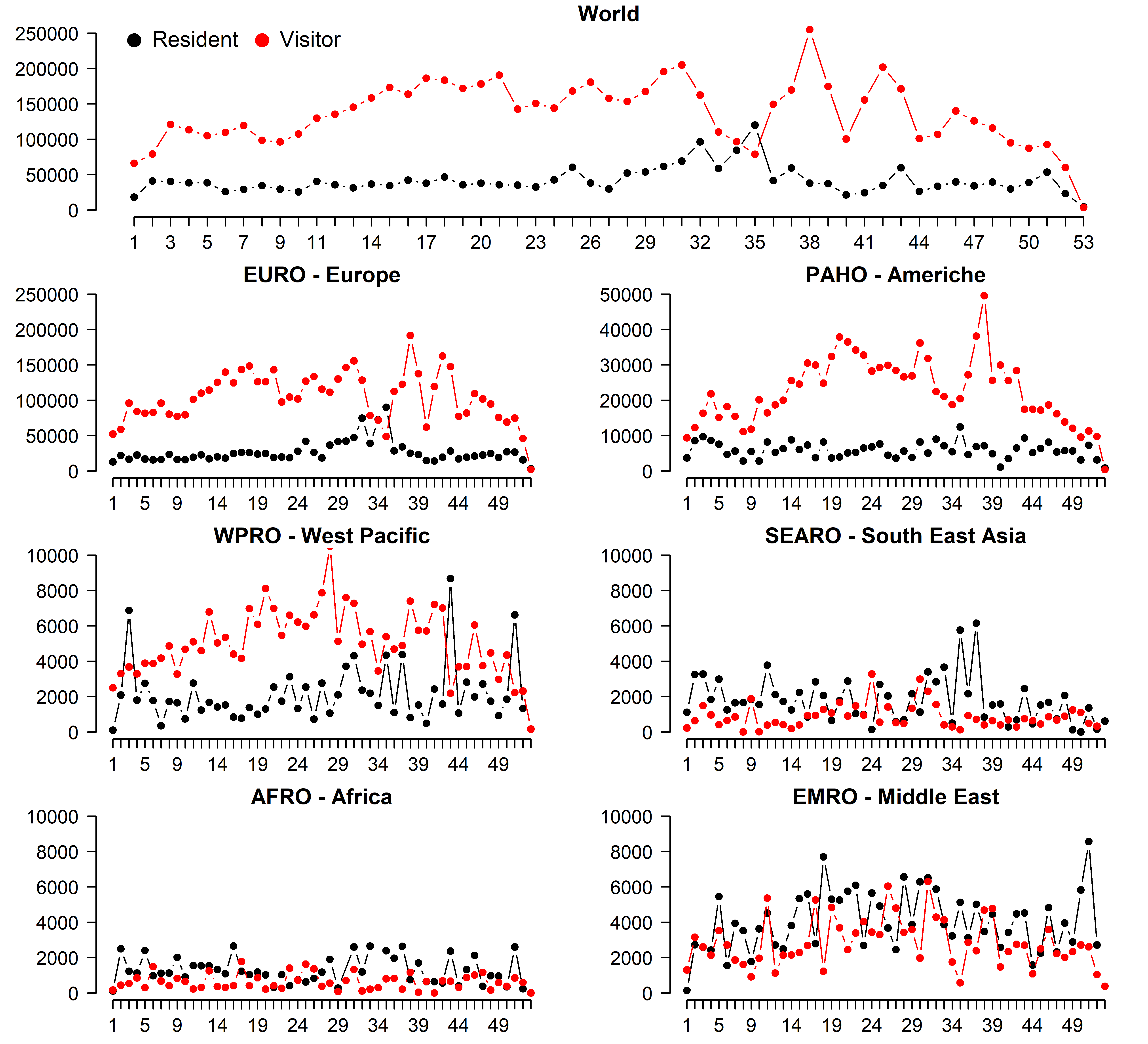


**Figure S4:** Weekly number of travellers (residents/visitors) that declared at least a one-night stay in Rome and their origin. On the x-axis the weeks of the year. Each panel represents a WHO region of provenience. On the y-axis the total number of travellers in the corresponding week. Black lines and dots represents residents travellers. Red lines and dots represents visitors travellers.
